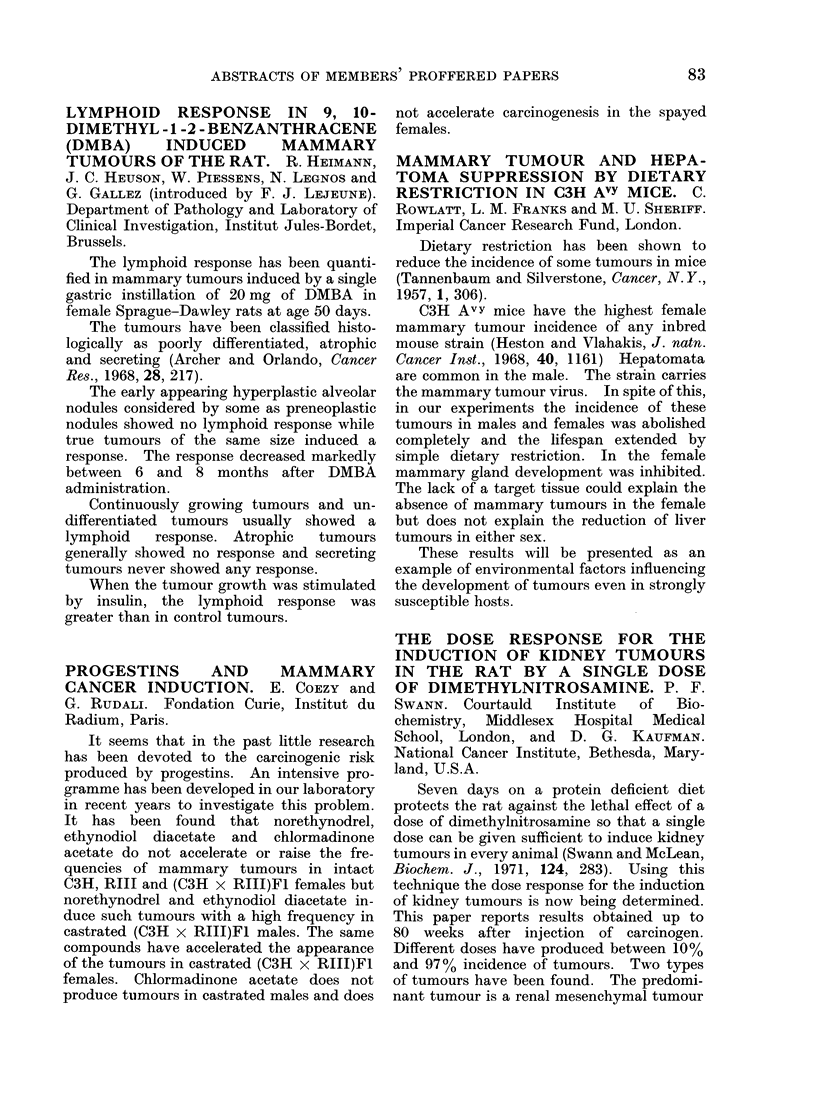# Progestins and mammary cancer induction.

**DOI:** 10.1038/bjc.1973.97

**Published:** 1973-07

**Authors:** E. Coezy, G. Rudali


**PROGESTINS AND MAMMARY CANCER INDUCTION.** E. COEZY and G. RUDALI. Fondation Curie, Institut du Radium, Paris.

It seems that in the past little research has been devoted to the carcinogenic risk produced by progestins. An intensive programme has been developed in our laboratory in recent years to investigate this problem. It has been found that norethynodrel, ethynodiol diacetate and chlormadinone acetate do not accelerate or raise the frequencies of mammary tumours in intact C3H, RIII and (C3H × RIII)Fl females but norethynodrel and ethynodiol diacetate induce such tumours with a high frequency in castrated (C3H × RIII)Fl males. The same compounds have accelerated the appearance of the tumours in castrated (C3H × RIII)Fl females. Chlormadinone acetate does not produce tumours in castrated males and does not accelerate carcinogenesis in the spayed females.